# Experimental study on seismic characteristics of slope supported by long-short composite anti-slide piles

**DOI:** 10.1038/s41598-024-65848-x

**Published:** 2024-07-02

**Authors:** Jie Lai, Yun Liu, Yuan Liu, Xu Jiangbo

**Affiliations:** 1 Xi’an Research Institute of High Technology, Xi’an, 710025 China; 2 School of Highway, Chang an University, Xi’an, 710064 China

**Keywords:** Natural hazards, Geodynamics, Seismology

## Abstract

In this study, a shaking table test was conducted on long-short composite anti-slide piles, the development process and dynamic response of cracks in a pile-supported slope were observed, and the failure mechanism of the slope was explored. The experiment showed that the failure of the pile-supported slope under an earthquake was a gradual process; cracks first occur at the top of the slope, where the support action of the piles was weak. As the input seismic action increased, cracks developed downwards along the slope. Owing to the support effect of the long-short anti-slide composite piles, the transmission path of the cracks changed, and the cracks developed along the top of the composite piles, ultimately leading to overtop failure. When cracks appeared on the slope or near final failure, the acceleration response law of the supported slope undergone a sudden change, which was an important indicator of slope instability. The distribution of dynamic soil stress on the pile body was greatly affected by the input peak ground acceleration, and the maximum bending moment of the long-short composite anti-slide piles was located near the weak interlayer.

## Introduction

Anti-slide piles are effective slope-support structures. When the landslide thrust is too large, single row piles are unable to withstand the entire sliding thrust. Long–short composite anti-slide piles are used to resist landslide thrust owing to their advantages, such as high overall stiffness, high lateral displacement resistance, strong anti-slide ability, and reasonable stress. Currently, numerical simulations, testing, and theoretical derivations are the common methods used to study the dynamic characteristics and stability of anti-slide piles.

Jia^1^ proposed a dynamic calculation method for rock slope stability considering anchor rods, and established the seismic degradation effect of rock anchor slopes. Nazari^2^ adopted a numerical sliding model to calculate the displacement of pile-reinforced slopes based on the results of numerical simulation and statistical analysis. Huang^3^ presented the results of a series of 50 g dynamic centrifuge tests that replicated the seismic response of pile-anchor reinforced slopes exposed to seismic stress fields. Chen^4^ investigated the dynamic response of a pile-anchor structure reinforcement for the slope with considering factors such as the acceleration field, the peak dynamic earth pressure on the piles, the deformation of the piles, the tension of the anchor cables, and the load sharing ratio. Sharafi^5^ established small-scale physical models and three-dimensional numerical models on a biaxial shaking table, and conducted combinations of one-dimensional, two-dimensional, and three-dimensional seismic loads on pile-stabilized slopes. Yu^6^ used a series of centrifuge model tests to study the dynamic behavior of pile-reinforced slopes subjected to various motions, and found that the overall response of the pile-reinforced slope was lower than that of the non-reinforced slope. Fan^7^ used shaking table test on a slope reinforced by double-row anti-slide piles and pre-stressed anchor cables to acquire the development of slope stability. Wang and Zhang^8^ conducted centrifuge model tests on pile-reinforced and unreinforced clay slopes to investigate the fundamental behavior and reinforcement mechanism, and established a finite element analysis model to compare its predictions with experimental results. Abi^9^ used the strength reduction method to obtain the stress distribution of anti-slide piles and the dynamic stability of the slope, and determined the final failure position of the slope in earthquakes. Ellis^10^ investigated the influence of pile spacing on the forces acting on anti-slide piles, and obtained the lateral pressure distribution, shear force, and bending moment of anti-slide piles with different pile spacings. Liao and Zhang^11^ used numerical analysis to analyze the effectiveness of the slope treatment from three aspects: slope stability safety factor, slope displacement deformation, and shear strain. Lai^12^ used a simplified analytical model to analyze a slope supported by double-row piles. Shen^13^ used the Winkler elastic foundation beam model to consider the deformation coordination relationship between the loaded and embedded sections, and provided a calculation method for the landslide thrust. Yang^14^ conducted a study on the calculation method and optimization design of long-short composite piles, using the finite element strength reduction method to analyze the stress characteristics of the piles. Al Defae^15^ used theoretical derivation combined with experimental verification to provide the mechanical performance of a slope supported by single row anti-slide pile under earthquakes, analyzed the relationship between the dynamic shear modulus of soil materials and the pile burial depth, and explored the maximum bearing capacity of the piles.

However, the above-mentioned research mainly focused on the static action situation, and there is relatively little research conducted on dynamic analysis; therefore, it cannot effectively reveal the dynamic response law and failure mechanism of pile-reinforced slopes under earthquakes. In response to these shortcomings, this study used shaking table tests to elucidate the failure process of a slope supported by long-short composite piles under earthquakes and revealed the dynamic response and failure mechanism of the slope. The test results provide a reference for the seismic design of long–short composite piles.

## Similar ratio

### Similarity derivation

Generally, the physical state of any geotechnical structure is represented as:

1 $$ f(l,\rho ,g,\sigma ,\tau ,t,E,I,G,c,w,p,M,v_{s} ,u,m) = 0 $$

where $l$ represents the geometric dimension; $t$ represents time; $p$ is a concentrated load; $g$ is the gravitational acceleration; $\sigma$ is the stress borne by the material; $\tau$ is the shear stress; $\rho$ is the material density; *E* is the elastic modulus; $I$ is the moment of inertia for the structure; *G* is the shear modulus; $c$ is the cohesion of the material; $w$ is the circular frequency; $T$ is the period; *p* is the concentrated load borne by the structure; $M$ is the bending moment under the action of external forces on the structure (such as earthquake action); $v_{s}$ is the shear wave velocity; *u* is the displacement of the particle; and $m$ is the material quality.

In the shaking table test, the gravitational acceleration of the model was consistent with that of the prototype (i.e.,), and the gravitational acceleration can be considered as the basic physical quantity. Finally, the length, gravitational acceleration, and material density were selected as the basic physical quantities. Based on the derivation process of dimensional analysis method (Kokusho, 2005,^17^, Shinoda^19^, it can be obtained that:

2 $$ \pi_{1} = \frac{\sigma }{lg\rho },\;\pi_{2} = \frac{\tau }{lg\rho },\;\pi_{3} = \frac{t}{{l^{0.5} \rho g}} $$

3 $$ \pi_{4} = \frac{E}{lg\rho },\;\pi_{5} = \frac{I}{{l^{4} }},\;\pi_{6} = \frac{G}{l\rho g} $$

4 $$ \pi_{7} = \frac{c}{\rho lg},\;\pi_{8} = \frac{w}{{l^{0.5} \rho g}},\;\pi_{9} = \frac{p}{{l^{3} g\rho }} $$

5 $$ \pi_{10} = \frac{M}{{\rho gl^{4} }},\;\pi_{11} = \frac{{v_{s} }}{{\rho gl^{0.5} }},\;\pi_{12} = \frac{u}{l},\;\pi_{13} = \frac{m}{{l^{3} \rho }} $$

where $l$ is the length; $v$ is the shear wave velocity; $\sigma$ is the stress; $t$ is the time; $E$ is the elastic modulus; $g$ is the gravitational acceleration; $\rho$ is the density; and $\lambda$ is the similitude ratio.

$\pi_{1}$ ~ $\pi_{10}$ are dimensionless quantities, and $(\pi_{i} )_{m} = (\pi_{i} )_{p}$, the following equations are established:

6 $$ C_{\sigma } { = }C_{l} C_{g} C_{\rho } ,\;C_{\tau } { = }C_{l} C_{\rho } C_{g} ,\;C_{t} { = }C_{l}^{0.5} C_{\rho } C_{g} $$

7 $$ C_{E} { = }C_{l} C_{\rho } C_{g} ,\;C_{I} { = }C_{l}^{4} ,\;C_{G} { = }C_{l} C_{\rho } C_{g} $$

8 $$ C_{c} { = }C_{\rho } C_{l} C_{g} ,\;C_{t} { = }C_{l}^{0.5} C_{\rho } C_{g} ,\;C_{p} { = }C_{l}^{3} C_{\rho } C_{g} $$

9 $$ C_{M} { = }C_{g} C_{\rho } C_{l}^{4} ,\;C_{{v_{s} }} { = }C_{\rho } C_{g} C_{l}^{0.5} ,\;C_{u} { = }C_{l} ,\;C_{m} { = }C_{l}^{3} C_{\rho } $$

Materials in the plastic stage can be expressed using the Mohr–Coulomb criterion.

10 $$ \tau_{f} { = }\sigma \tan \varphi { + }c $$

where $\tau_{f}$ denotes the shear strength, $\sigma$ denotes the normal stress, $c$ denotes the cohesion, and $\varphi$ denotes the internal friction angle. To obtain similarity in the limit state, the final similarity relationship should satisfy the following conditions:

11 $$ C_{\varphi } { = }1,\;C_{c} { = }C_{l} C_{\rho } C_{{\text{g}}} $$

### Similar ratio selection

However, the dimensional analysis method also has following limitations:

1. It is not able to display the influence of each physical quantity on the test results.
2. It is difficult to distinguish the key points of selection of similarity ratio relationships for different materials.
3. In the dimensional analysis method, the materials in the experiment must satisfy the similarity relationship listed in Table 1, which is extremely difficult to fulfill, and the experiment is difficult to be conducted. Therefore, it is necessary to make certain trade-offs in the similarity relationships that the material must satisfy based on the test purpose (Lin^20^).

**Table 1:** Main similarity constant of the model.

Physical quantity	Similarity relation	Similarity constant	Physical quantity	Similarity relation	Similarity constant
density	$C_{\rho }$	1	internal friction angle	$C_{\varphi } = 1$	1
length	$C_{l}$	20	stress	$C_{\sigma } = C_{c} = C_{\rho } C_{l} C_{g}$	20
elastic modulus	$C_{E} = C_{\rho } C_{l} C_{g}$	20	time	$C_{t} = C_{l}^{0.5} C_{\rho } C_{g}$	4.472
displacement	$C_{{\text{u}}} = C_{l}$	20	frequency	$C_{f} = 1/C_{t}$	0.224
acceleration	$C_{a} = C_{{\text{g}}}$	1	shear wave velocity	$C_{{v_{s} }} = C_{l}^{0.5} C_{\rho } C_{g}$	4.472
bending moment	$C_{M} { = }C_{g} C_{\rho } C_{l}^{4}$	20^4^	force	$C_{p} { = }C_{l}^{3} C_{\rho } C_{g}$	20^3^

The similarity ratio in the table refers to the ratio of the physical quantities corresponding to the prototype and the model.

## Model test

### Basic overview of the test

**Figure 1:**
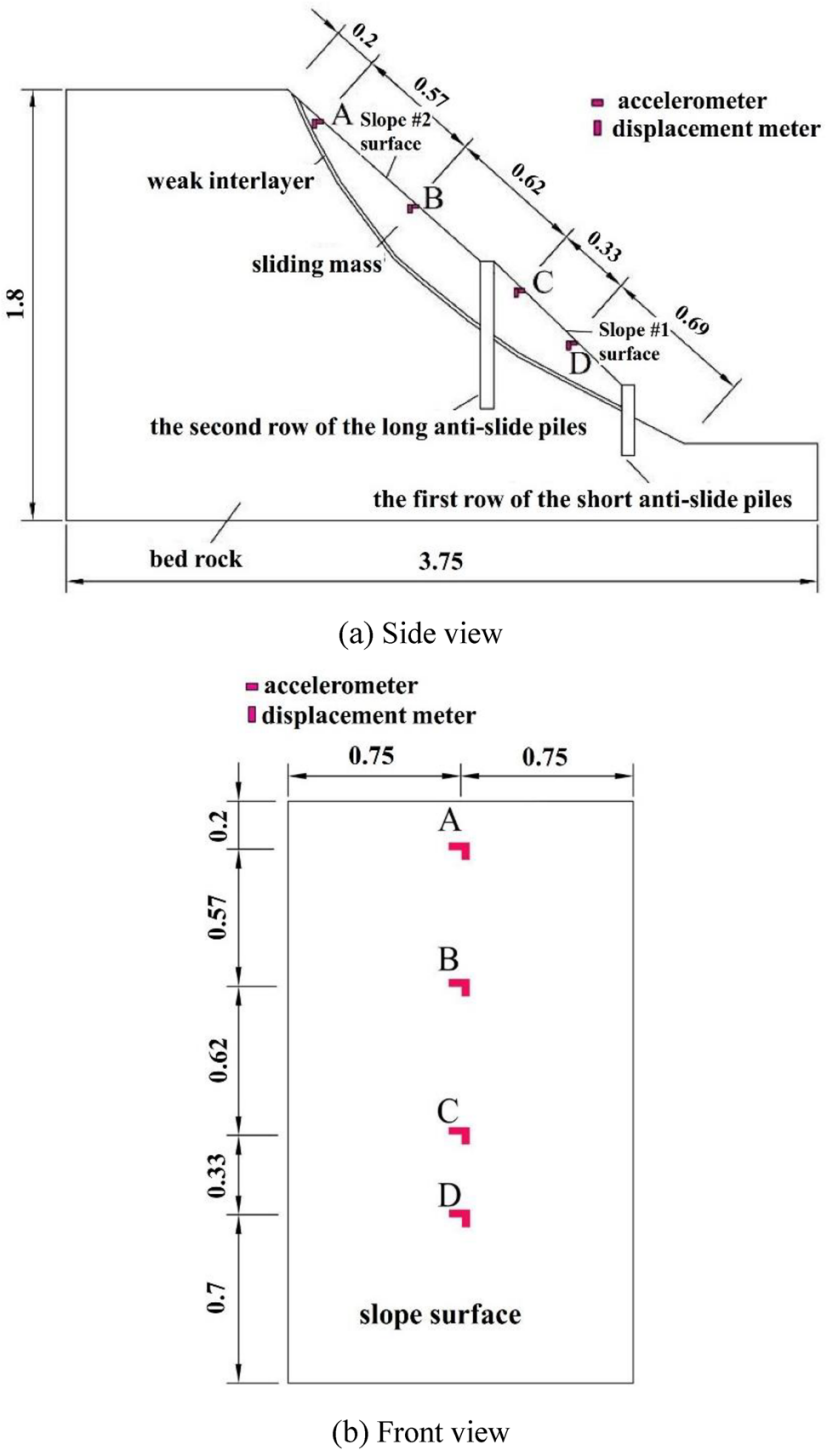
Slope model diagram (unit: m).

The shaking table test in this study simulated a slope with a height of 36 m composed of an upper sliding mass (It is the main component of a landslide, which refers to the rock and soil mass that slides down a slope. The depth of sliding mass was from 0 to 0.28 m), a weak interlayer (the thickness of the weak interlayer was about 0.015 m), and a lower bedrock. The slope surface was divided into two parts by the composite anti-slide piles. The slope surface between the two piles was called slope #1 and the slope surface adjacent to the top of the slope was called slope #2. The cross-sectional size of the anti-slide pile is 1.2 m × 1.6 m, with a pile spacing of 5 m. The lengths of the first and second rows of anti-slide piles were 7 m and 13 m, respectively. To fulfill the size similarity ratio relationship in Table 1, the height of the slope model in the experiment was 1.8 m, and the pile section was set at 0.06 m × 0.08 m, with a pile spacing of 0.25 m. The first row of piles was 0.35 m long, and the second row of piles is 0.65 m long. The material of long-short composite anti-slide piles was glued with rigid plastic boards, and the elastic modulus of each plastic board was 1500 MPa. The layout is shown in Fig. 1.

**Figure 2:**
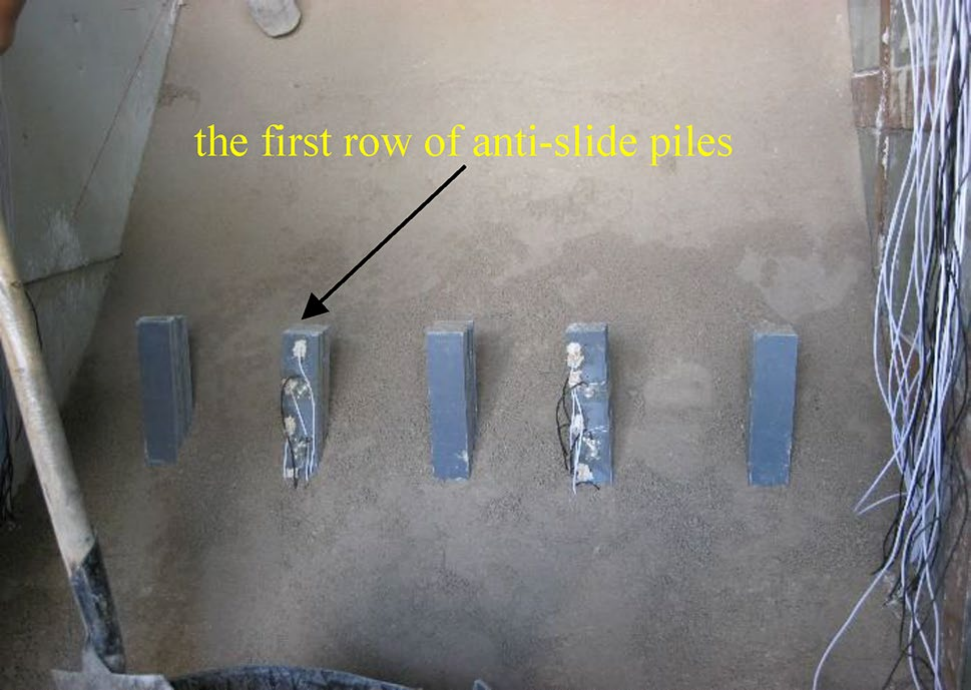
Schematic diagram of anti-slide piles.

Standard sand, gypsum powder, talc powder, glycerol, cement, and water were used as the basic materials in the test. The internal friction angle of the standard sand was 32°, and the cohesive force was 0 kPa. The particle size of talc powder was 12.5 μm. The density of glycerol was approximately 1.26 g/cm^3^ and the strength grade of cement was 42.5 Mpa. The material parameters were determined through relevant experiments, and the final mix proportions and material parameters were selected as listed in Table 2. The model material was controlled by the density and placed in a model box (as shown in Fig. 2.)

**Table 2:** Physical and Mechanical Parameters of Materials.

Type		Elastic modulus/MPa	Friction angle/°	Cohesive force/kPa	Density kg/m^3^
Sliding mass		20	33	18	1950
Weak interlayer		6	28.5	4	1830
Bed rock		53	39	30	2030

Based on the experimental data, the physical and mechanical parameters of each material layer were listed in Table 2.

Four horizontal accelerometers (the accelerometers was of 0–20 g, with an accuracy of 0.005 g) and four horizontal displacement meters (the horizontal displacement meters was of 0-1m, with an accuracy of 0.001 m) were arranged on the model slope, represented as A, B, C, and D. The specific locations were shown in Fig. 1. The working frequency of the accelerometer was of 0.1–100 Hz. The horizontal displacement sensor recorded the displacement relative to the vibration table with a resolution of 0.1 mm.

**Figure 3:**
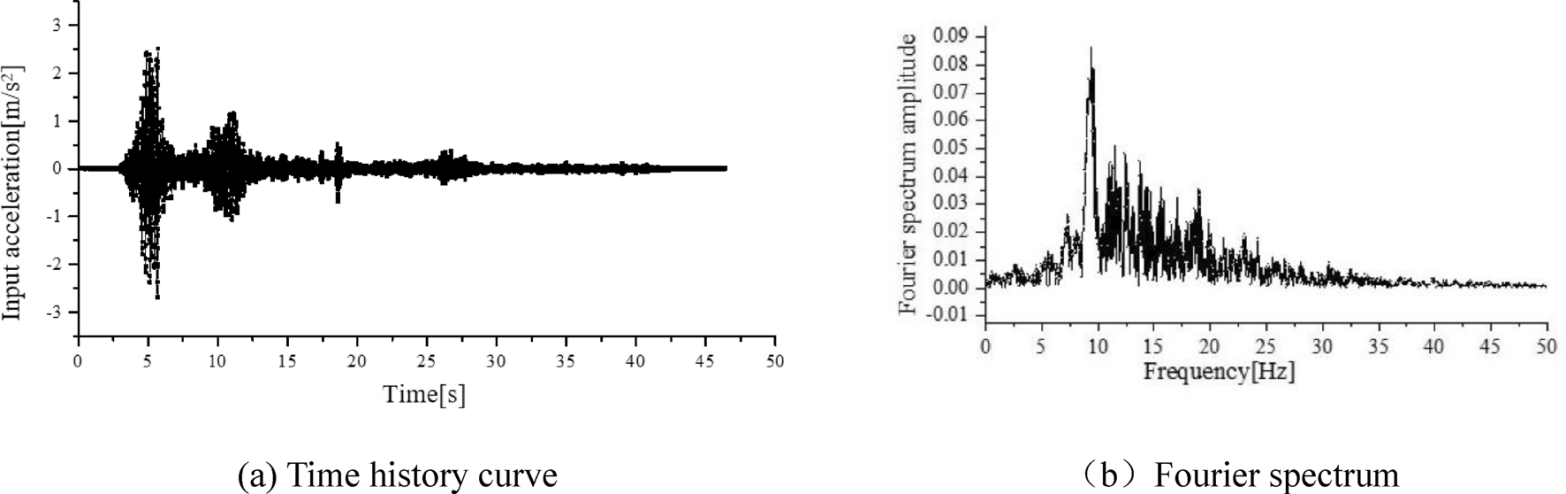
Input horizontal seismic acceleration curve.

In this experiment, representative Wenchuan seismic waves were selected as the excitation source. To explore the impact of seismic intensity, the peak acceleration of each input seismic wave was adjusted, starting from 0.1 g and gradually loading, with each level being increased by 0.1 g until it reached 1.0 g. The input ground acceleration was bidirectional with horizontal seismic waves along the slope direction. According to statistical data, the peak ratio of the vertical to horizontal seismic acceleration during the Wenchuan earthquake was close to 2/3 (Luo et al.^21^. Therefore, the experimental vertical peak acceleration was loaded 2/3 times the horizontal value. The horizontal waveform obtained after compression at a time compression ratio of 1: $\sqrt {20}$ was shown in Fig. 3.

### Test results

**Figure 4:**
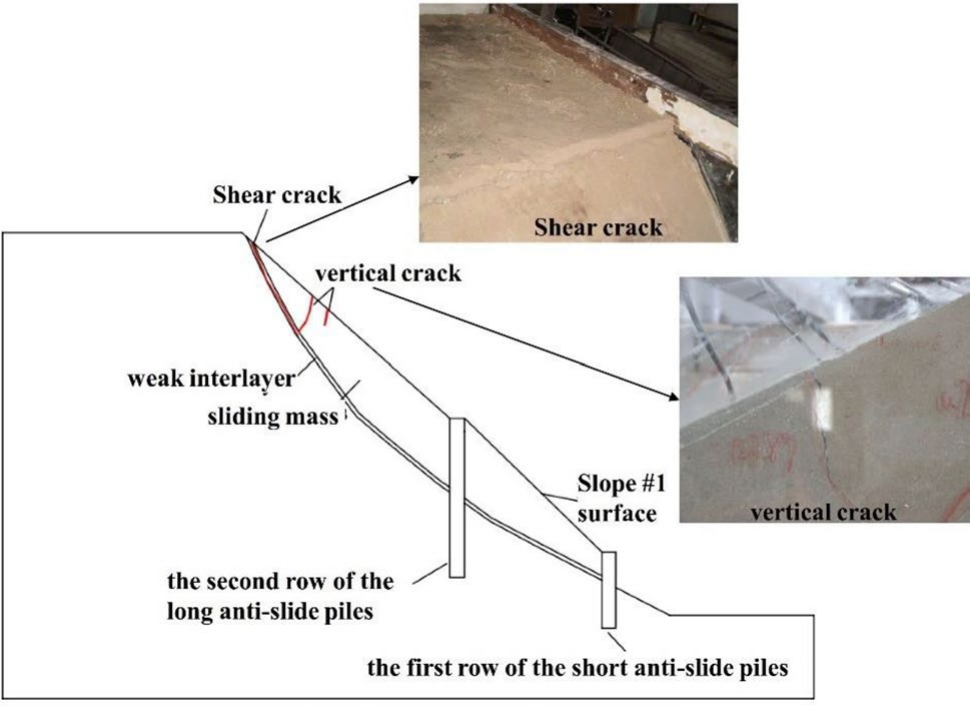
Failure state diagrams of the model after earthquake under different peak accelerations.

There was no significant change on the slope surface when the input peak ground acceleration (PGA) changed from 0.1 to 0.3 g. When PGA = 0.4 g (Fig. 4), a transverse shear crack with a width of 1–2 mm first appeared at the top of slope #2 near the sliding surface. The occurrence of cracks indicated that the slope has entered the failure process. The cracks generated at PGA = 0.4 g were mainly shear cracks, which would propagate downwards along the weak interlayer. Owing to the support effect of the composite anti-slide piles, the supported slope still had bearing capacity, and there was no overall damage to the slope at this time. When the PGA reached 0.6 g, a significant displacement occurred in the weak interlayer near the top of the slope. In addition to the shear cracks at the top of the slope developing downwards along the weak interlayer, some vertical cracks appeared in the middle of slope #2, with a width of 3–4 mm.

**Figure 5:**
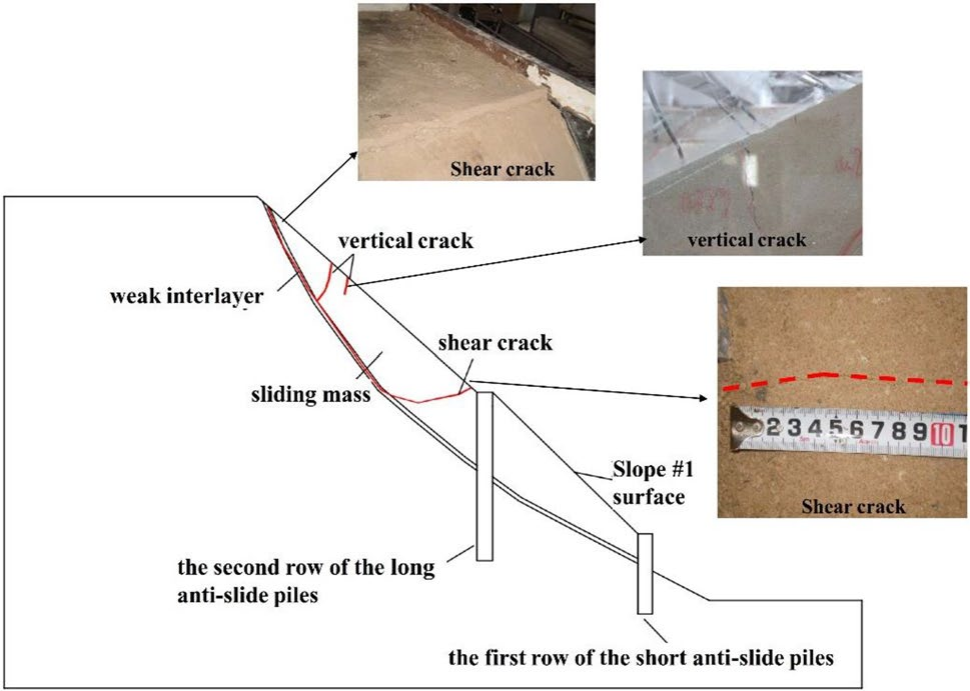
Dynamic failure surface of the slope (side view).

When PGA changed from 0.7 to 0.9 g (as shown in Fig. 5), the shear crack generated at PGA = 0.4 g propagated downwards along the weak interlayer and stop expansion near the second row of anti-slide piles. Besides, a transverse shear crack appeared on the slope surface at the foot of the slope #2, with a width of 0.3–0.5 mm. This crack was also located at the top of the second row of the anti-slide piles, indicating that it had already been connected with the shear crack from the weak interlayer, and the slope had undergone overtop failure.

The above analysis revealed the failure process of the long-short composite pile-reinforced slope. That was, when the PGA reached 0.4 g, cracks first appeared at the weak interlayer at the top of the slope. It was because that the weak interlayer at the top of the slope was very steep, and the sliding mass slid down along the weak interlayer, resulting in the occurrence of cracks at the top of the slope. When the PGA reached 0.6 g, the cracks started to develop downward along the weak interlayer and stop expansion near the second row of anti-slide piles with the increase in PGA, and several vertical cracks were also generated in the upper part of the slope. Among them, the top vertical crack was connected to the shear crack at sliding surface. When the PGA reached 0.9 g, the transverse crack generated at the foot of the slope #2 intersected with the existing shear crack at sliding surface. Owing to the support effect of the composite piles, the shear crack changed its development direction as it approached the pile body, and the sliding surface eventually slid out over the top of the second row of anti-slide piles. Consequently, a transverse crack appeared on the slope surface above the top of the second row of anti-slide piles, which caused the overall failure of the slope. The cracks generated at each stage were summarized in Fig. 5.

From the failure phenomenon of the long-short composite pile-reinforced slope, it could be observed that the failure mainly occurred on slope #2, whereas there were no cracks on slope #1. The test showed that slope #2 should be separately reinforced to improve the seismic resistance of the reinforced slope.

**Figure 6:**
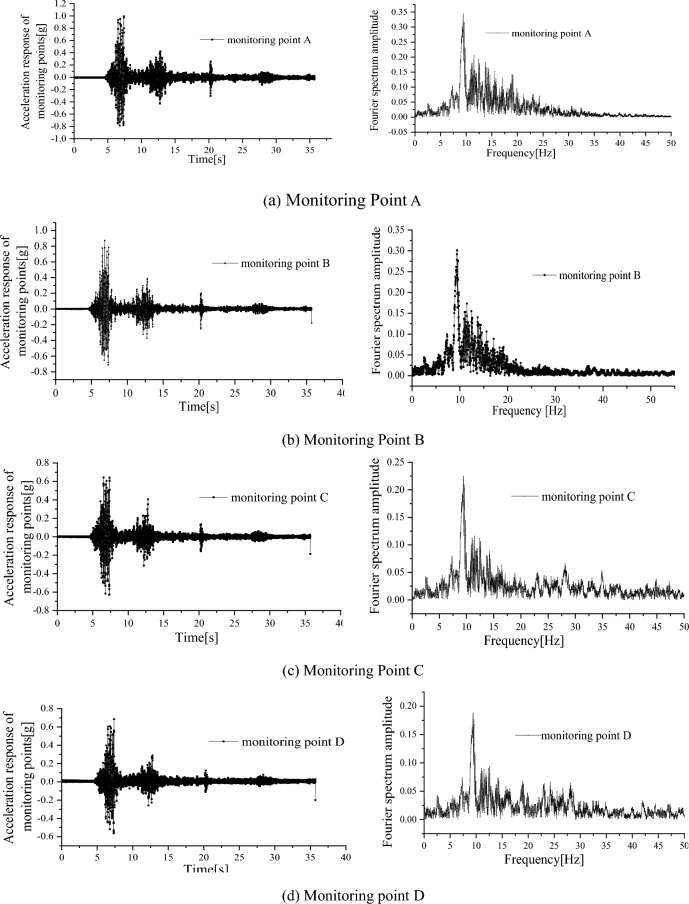
Acceleration response curve of monitoring points in no anchor side (0.4 g, Wenchuan).

Figure 6 shows the acceleration response time history curve of the monitoring points on the slope when the PGA was 0.4 g. It could be observed that the peak response acceleration at each monitoring point was greater than that of the input seismic wave, indicating that there was a certain acceleration amplification effect on the slope. The acceleration response of monitoring point A was the most evident response, with an acceleration amplification coefficient of approximately 2.34 times. The response at monitoring point D was the smallest, with an acceleration amplification coefficient of 1.73 times. From the Fourier spectra of monitoring points, it can be noted that the seismic response spectrum components of the slope surface were mainly concentrated in the range of 2–18 Hz, with the most evident response around 10 Hz. After 20 Hz, the higher the position of the monitoring point, the smaller the Fourier spectrum amplitude. At 10 Hz, the higher the monitoring point position, the greater its Fourier amplitude. The data show that the slope has the characteristics of low-frequency amplification and high-frequency filtering of the input seismic waves.

**Figure 7:**
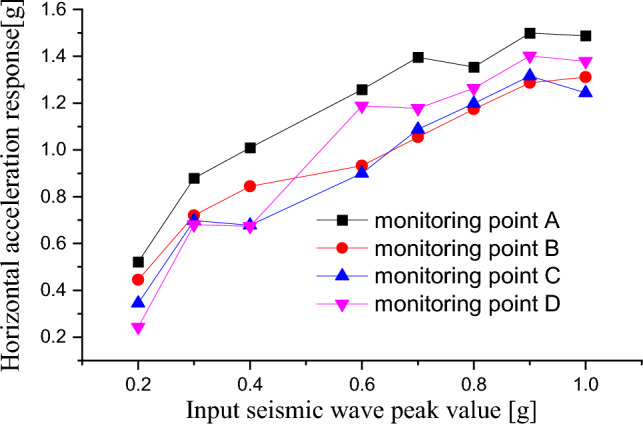
Response curve of acceleration and peak value of seismic wave.

Figure 7 shows the peak acceleration values of the monitoring points under different earthquake conditions. As the earthquake intensity increased, the acceleration response at each monitoring point became more evident. When the PGA changed from 0.2 to 0.4 g, the higher the position of the monitoring points on the slope, the greater the acceleration response. When the PGA was above 0.4 g, there was a sudden change in the slope acceleration response, and the acceleration magnitude of monitoring point D exceeded that of monitoring point B. It was because that cracks have appeared on the slope when the PGA was above 0.4 g. When the PGA was 1.0 g, it experienced a sudden change again with a decrease in response acceleration, indicating that the slope has been severely damaged. It was apparent from the test that before slope failure, the acceleration response of the slope increased with the increase of the input peak ground acceleration (PGA). However, when approaching failure, due to the generation of the cracks, the acceleration response of the slope changed significantly. The acceleration response of the slope did not increase with the increase of the PGA, and even decreased.

**Figure 8:**
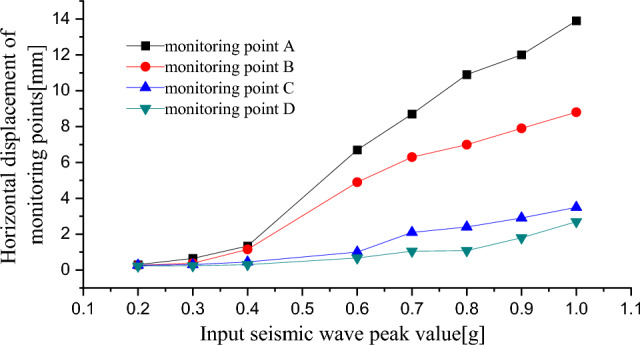
Response curve of displacement and peak value of seismic wave.

Figure 8 shows the relationship between the PGA and cumulative displacement of the monitoring points during the earthquakes. When 0.2 g ≤ PGA ≤ 0.3 g, the horizontal displacement of the monitoring points was mainly relatively small, elastic deformation. When 0.4 g ≤ PGA ≤ 1.0 g, the displacement of monitoring points rapidly developed, and the cumulative deformation increased. Plastic deformation was the main type of deformation. The displacement changed at the monitoring points were consistent with the above-mentioned development of cracks.

**Figure 9:**
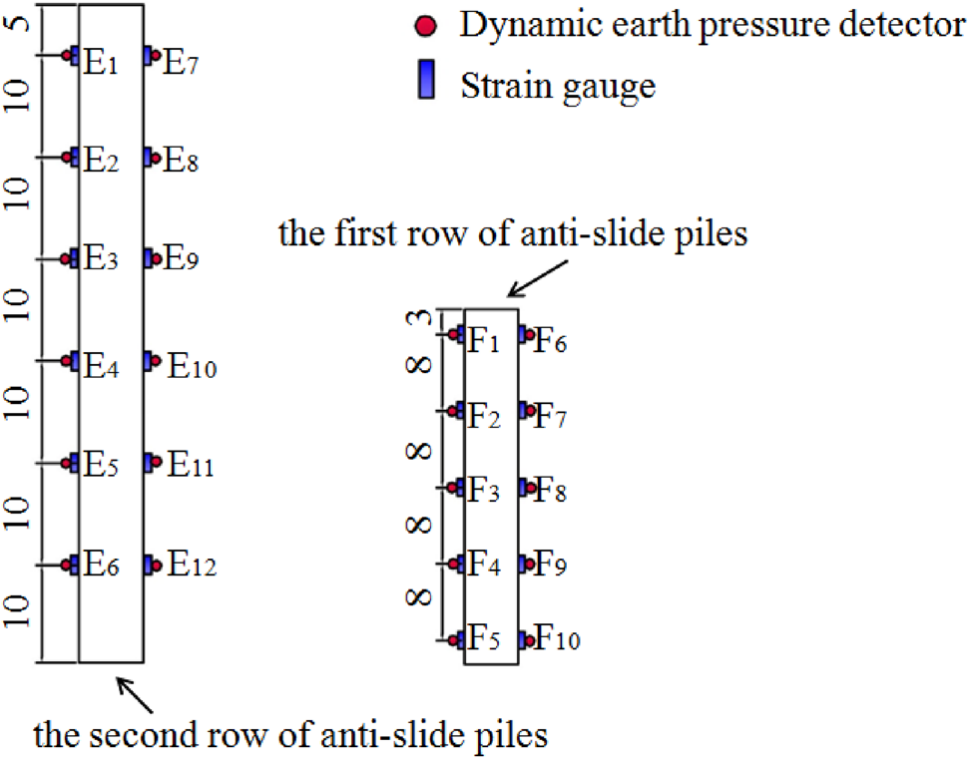
Schematic diagram of the location of strain gauges for anti-slide piles (unit cm).

To obtain the internal force distribution of the anti-slide piles under earthquake conditions, strain gauges and dynamic soil pressure boxes were installed on both sides of the anti-slide piles, shown as F1–F10 and E1–E12, respectively in Fig. 9. F1–F5 and E1–E6 were located near the upslope surface of the piles.

When calculating the internal force of the anti-slide piles, the bending moment and axial force of the piles were obtained using Eq. (12) based on the recorded data form the installed strain gauges. The internal force of the structure also changed owing to the continuous variation in earthquake action. To better guide engineering practices and ensure structural safety, this study statistically analyzed the peak value of the internal force of anti-slide piles under different earthquake intensities.

12 $$ M = (\varepsilon_{1} - \varepsilon_{2} )E_{c} W/2 = (\varepsilon_{1} - \varepsilon_{2} )E_{c} bh^{2} /12 $$

where $\varepsilon_{1}$ and $\varepsilon_{{2}}$ are the strain values on both sides of the anti-slide pile, $E_{c}$ is the converted elastic modulus of the pile, $h$ is the height of the anti-skid pile, and $b$ is the width of the pile.

**Figure 10:**
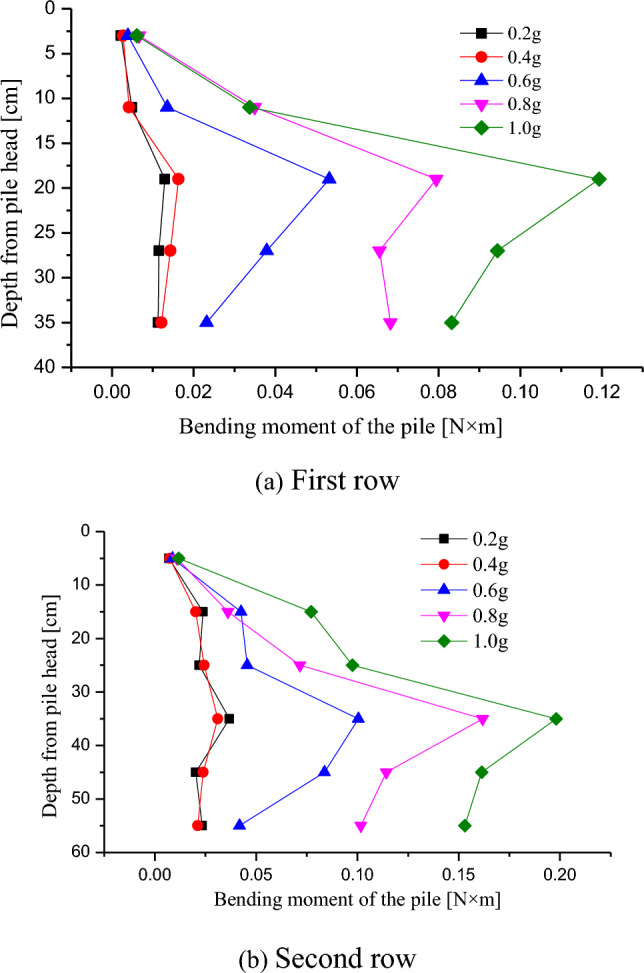
Moment distribution of the anti-slide piles.

From the distribution of the bending moments of the first and second rows of the anti-slide piles in Fig. 10, it could be observed that the distribution of the bending moments of the piles under an earthquake was approximately parabolic, reaching its maximum value at the lower position near the weak interlayer. The PGA has a significant impact on the bending moment, and the bending moment of the anti-slide piles increased nonlinearly with an increase in the PGA. In the same situation, the bending moment of the second row of anti-slide piles was greater than that of the first row of anti-slide piles. The main reason for this was that the second row of the anti-slide piles had a longer pile body and was closer to slope #2. The sliding force of the sliding mass on slope #2 was mainly borne by the second row of piles, whereas that of the slope #1 was mainly borne by the first row of piles. From the variation of bending moment values, when PGA ≤ 0.4 g, the bending moment of anti-slide piles was relatively slow; when PGA > 0.4 g, the bending moment of the anti-slide piles increased rapidly, and the second row of the anti-slide piles bear a greater bending moment than the first row of the anti-slide piles.

## Conclusion

1. Under the action of earthquakes, slope failure is a gradual process and the fracture surface of the sliding mass is mainly composed of tensile and shear cracks. According to this experiment, slope cracks occur at the top of Slope #2 near the sliding surface firstly. As the action of earthquakes increases, cracks develop downward along the weak interlayer and change the direction of development near the anti-slide piles.
2. As the seismic effect increases, the acceleration response at each monitoring point becomes more evident. Generally, the higher the position of the monitoring point on the slope, the greater the acceleration and displacement responses. However, when cracks occur in the slope and the slope approaches a failure state, the acceleration response exhibits anomalies, and the displacement of the slope rapidly increases.
3. The force analysis of the anti-slide piles shows that the landslide thrust is mainly borne by the second row of piles near the top of the slope. When PGA < 0.4 g, the development of slope cracks is still in the initial stage, and the peak growth of the bending moment of the anti-slide piles is relatively slow. When PGA ≥ 0.4 g, the development of slope cracks is rapid, and the bending moment of the anti-slide piles increases exponentially, following a non-linear pattern.

## Data Availability

The data used to support the findings of this study are available from the corresponding author upon request.
